# The effects of macronutrients composition on hormones and substrates during a meal tolerance test in drug-naive and sitagliptin-treated individuals with type 2 diabetes: a randomized crossover study

**DOI:** 10.20945/2359-3997000000478

**Published:** 2022-05-12

**Authors:** Cristina da Silva Schreiber, Alex Rafacho, Renata Silverio, Roberto Betti, Antonio Carlos Lerário, Ana Maria Pita Lotenberg, Klara Rahmann, Carolina Piras de Oliveira, Bernardo Léo Wajchenberg, Protásio Lemos da Luz

**Affiliations:** 1 Universidade de São Paulo Faculdade de Medicina Instituto do Coração São Paulo SP Brazil Instituto do Coração (InCor), Faculdade de Medicina, Universidade de São Paulo (USP), São Paulo, SP, Brasil; 2 Universidade Federal de Santa Catarina Centro de Ciências Biológicas Departamento de Ciências Fisiológicas Florianópolis SC Brazil Laboratório de Investigação em Doenças Crônicas (LIDoC), Departamento de Ciências Fisiológicas, Centro de Ciências Biológicas, Universidade Federal de Santa Catarina (UFSC), Florianópolis, SC, Brasil; 3 Universidade de São Paulo Faculdade de Medicina Hospital das Clínicas São Paulo SP Brazil Laboratório de Lipídios (LIM10), Hospital das Clínicas, Faculdade de Medicina, Universidade de São Paulo (USP), São Paulo, SP, Brasil; 4 Universidade de São Paulo Faculdade de Medicina Hospital das Clínicas São Paulo SP Brazil Hospital das Clínicas, Faculdade de Medicina, Universidade de São Paulo (USP), São Paulo, SP, Brasil

**Keywords:** Diabetes, diet, glycemia, glucose tolerance, incretins, meal tolerance test

## Abstract

**Objectives:**

To evaluate the effect of sitagliptin treatment in early type 2 diabetes mellitus (T2DM) and the impact of different macronutrient compositions on hormones and substrates during meal tolerance tests (MTT).

**Materials and methods:**

Half of the drug-naive patients with T2DM were randomly assigned for treatment with 100 mg of sitagliptin, q.d., or placebo for 4 weeks and then submitted to 3 consecutive MTT intercalated every 48 h. The MTTs differed in terms of macronutrient composition, with 70% of total energy from carbohydrates, proteins, or lipids. After 4 weeks of washout, a crossover treatment design was repeated. Both patients and researchers were blinded, and a repeated-measures ANOVA was employed for statistical analysis.

**Results:**

Sitagliptin treatment reduced but did not normalize fasting and post-meal glucose values in the three MTTs, with lowered area-under-glucose-curve values varying from 7% to 15%. The sitagliptin treatment also improved the insulinogenic index (+86%) and the insulin/glucose (+25%), glucagon-like peptide-1/glucose (+46%) incremental area under the curves. Patients with early T2DM maintained the lowest glucose excursion after a protein- or lipid-rich meal without any major change in insulin, C-peptide, glucagon, or NEFA levels.

**Conclusion:**

We conclude that sitagliptin treatment is tolerable and contributes to better control of glucose homeostasis in early T2DM, irrespective of macronutrient composition. The blood glucose excursion during meal ingestion is minimal in protein- or fat-rich meals, which can be a positive ally for the management of T2DM. Clinical trial no: NCT00881543

## INTRODUCTION

Diabetes mellitus (DM) is one of the most prevalent chronic diseases worldwide, and the incidence of this disease in developing countries has increased remarkably, in part due to the introduction of modern lifestyles, which often include the consumption of ultra-processed foods and a low degree of activity (1). Type 2 DM (T2DM) relies on a continuous improvement of lifestyle and treatment with oral antidiabetic drugs. Sitagliptin is a class of oral drugs available for the treatment of T2DM that was approved by the US Food and Drug Administration (FDA) in 2006 (2). Sitagliptin is a dipeptidyl peptidase-4 (DPP-4) inhibitor that acts by maintaining endogenous levels of glucagon-like peptide-1 (GLP-1) (3). GLP-1 potentiates the insulin secretion on beta cells that respond in a glucose-dependent manner (3). Alone or in combination with metformin, sitagliptin is effective in reducing fasting or postprandial blood glucose due to its action in preserving or increasing the endogenous incretins effect (4-7).

Blood glucose excursions after meals are known to have significant variability. They can be affected by the macronutrient composition of the specific meal and are dysregulated in individuals with T2DM in parts due to the reduced incretin effect (7-9). The acute benefit of sitagliptin on postprandial glycemia was demonstrated in healthy individuals after ingesting isocaloric glucose, olive oil, or protein mixture and involved augmented intact GLP-1 levels (6). However, individuals with impaired fasting glucose (IFG) who submitted to an 8-week treatment with sitagliptin had unchanged postprandial plasma glucose, insulin, or C-peptide, despite an increase in intact GLP-1 (10). Although numerous clinical studies have proven the benefit of sitagliptin in reducing glucose excursion after glucose or meal tolerance tests (MTT) (4,11,12), researchers need to clarify how different macronutrient composition meals could impact the effect of sitagliptin on glucose, insulin, C-peptide, glucagon and GLP-1 excursion in early T2DM. Thus, we evaluated the impact of sitagliptin treatment in naive-drug T2DM individuals and then submitted them to consecutive carbohydrate-, protein- or fat-rich MTTs.

## MATERIALS AND METHODS

### Ethics and clinical registration

This study consisted of a single-center, randomized, double-blinded, single-dose, placebo-controlled, and crossover prospective design. All participants provided written informed consent. The study was approved by the Research Ethics Committee of the University Hospital at the University of São Paulo (CEP-HU/USP 752/07 and 1117/13, CAAE 0031.0.198.019-07) and registered at ClinicalTrials.gov (NCT00881543, April 2009). All procedures followed the Declaration of Helsinki.

### Participants

Sixteen individuals from both sexes, aged between 40 and 70 years, were eligible to participate in the study. The sample size, based on a statistical power of 80% and type I error rate of 5%, was calculated *a priori* with the public sample size tool (http://powerandsamplesize.com). Participants were recruited from an external Diabetes campaign promoted and managed by the Heart Institute, School of Medicine, University of São Paulo. Participants had recently been diagnosed with diabetes (<3 months), confirmed by the standard 2-hour oral glucose tolerance test (OGTT) with 75 g of glucose and measurement of glycated hemoglobin (HbA1c) levels. Type 2 diabetes mellitus (T2DM) was confirmed when plasma glucose levels were higher than 200 mg/dL 2-hour after OGTT and HbA1c ≥ 6.5% (48 mmol/mol)

The inclusion criteria were as follows: drug-naive individuals with T2DM (individuals were considered drug-naive if they had never been treated with an oral antidiabetic agent or who had not taken any antidiabetic agent for at least 12 weeks before the study entry and never received antidiabetic agents for > 3 months at any time in the past); body mass index (BMI) from 20 to 35 kg/m^2^. Individuals were excluded from the study if they met any of the following criteria: comorbidities such as malignancies, liver or kidney disease, congestive heart failure; uncontrolled arterial hypertension; ongoing treatment with dipeptidyl peptidase-4 (DPP-4) inhibitors, growth hormone or similar, anti-arrhythmic, corticosteroid, proton-pump inhibitors or any other medication capable of altering gastric motility; hypolipidemic agents; acute diabetic decompensation, HbA1c > 10% (86 mmol/mol); positive for glutamic acid decarboxylase (GAD), insulin or islet cell antibodies; limiting psychiatric diseases; treatment with insulin or with a history of ketonuria or positive type 1 diabetes markers, and using other oral antidiabetic agents; pregnancy or breastfeeding; menopausal women using hormone replacement therapy; a history of active substance abuse within the past 2 years (including alcohol).

### Randomization and study design

**Figure 1:**
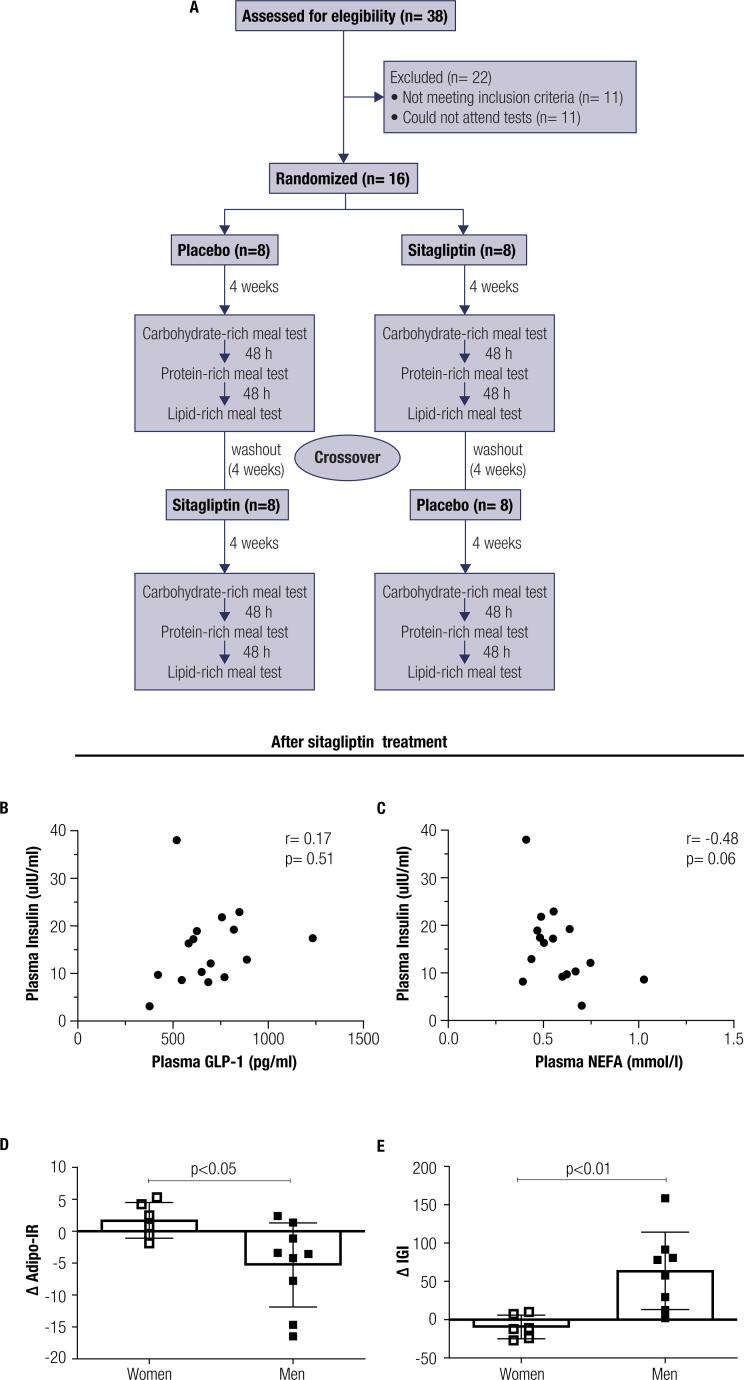
Flow chart and gender-specific sitagliptin effect. In **(A)** the flow chart of study participants used in the analysis. In **(B and C)** the lack of correlation between plasma insulin and plasma GLP-1 or NEFA, after 4 weeks of 100 mg sitagliptin treatment, *q.d.* In **(D)** the delta (Δ) values for the Adipo-IR (obtained in relation to the respective gender-placebo values) after sitagliptin treatment separated by gender. In **(E)** the Δ IGI (obtained in relation to the respective gender-placebo values) after sitagliptin treatment grouped by gender. A Pearson correlation test was applied in **(B, C)**. Data are mean ± SD in **(D, E)**. The statistical difference was obtained with an unpaired *t*-test **(D, E).** n = 16 for **(B, C)** and 6-7 (female) and 8-9 (male) for **(D, E)**. GLP-1, glucagon-like peptide-1; NEFA, non-esterified fatty acids; q.d. derives from Latin *quaque die* and means once daily; Adipo-IR, adipose tissue insulin resistance index, IGI, insulinogenic index.

Of 38 individuals, 16 were enrolled in the study program. Eleven individuals did not meet the inclusion criteria of the study, and 11 individuals could not attend the tests. The study patient disposition is presented in Figure 1A.

Participants were randomly allocated (lottery method) to initial treatment, *q.d.*, with 100 mg of sitagliptin (n = 8) (Januvia^®^, Merck Sharp Dome) or placebo (n = 8) (manufactured by the Clinic Hospital Pharmacy, University of São Paulo) before breakfast. All participants were treated with placebo and sitagliptin alternately according to a crossover design. Any adverse events were registered. After 4 weeks of treatment with either sitagliptin or placebo, participants were subjected to three different MTTs. Patients were told to present their empty medication blisters for verification. The MTTs were performed in 48-hour intervals between each test according to the following sequence: carbohydrate-rich meal (CHO), protein-rich meal (PTN), and lipid-rich meal (LIP) (Figure 1A). Patients coming from the sitagliptin branch remained on the sitagliptin treatment regimen up to the last meal test, while placebo-treated individuals received a placebo. They ingested the pill 1 hour before the meal test began. The period of drug washout before continuation of the cross-over design was 4 weeks. Then, the three MTTs were repeated at the end of treatment as described before.

### Meal tolerance tests

The three meals were isocaloric and consisted of similar solid consistency and fibers (Tables 1-4). The meals were formulated in collaboration with the Condieta^®^ company (São Paulo, SP, Brazil) to make them palatable and allow for their comparison.

**Table 1:** Energy and macronutrient composition of tested meals

	CHO	PTN	LIP
Energy (kcal)	450	465	469
Carbohydrate (g)	82	12.2	15.5
Carbohydrate (% of total energy)	**72**	10	13
Protein (g)	9.2	76.4	14
Protein (% of total energy)	8	**66**	13
Lipid (g)	9.5	12.2	39.5
Lipid (% of total energy)	20	24	**74**
Fiber (g)	5.2	4	4

CHO: carbohydrate-rich meal; PTN: protein-rich meal; LIP: lipid-rich meal.

**Table 2:** Food and macronutrient composition of the carbohydrate-rich meal

Food	Portion size (g)	Energy (kcal)	Protein (g)	Lipid (g)	Carbohydrate (g)	Fiber (g)
Rice	120	145	2	0.1	34	2.4
Potato	100	53	1.2	0.1	12	1.3
Pasta	50	174	6	0.7	36	1.5
Butter	5	37	-	4.1	-	-
Olive oil	4.5	41	-	4.5	-	-
Total	279.5	450	9.2	9.5	82	5.2

**Table 3:** Food and macronutrient composition of the protein-rich meal

Food	Portion size (g)	Energy (kcal)	Protein (g)	Lipid (g)	Carbohydrate (g)	Fiber (g)
Egg white	35	190	47	0.2	-	-
Egg yolk	15	52	2.4	4.6	0.2	-
Oat	20	70	-	2.4	12	4
Whey protein	27	108	27	-	-	-
Olive oil	5	45	-	5	-	-
Total	416	465	76.4	12.2	12.2	4

**Table 4:** Food and macronutrient nutrient composition of the lipid-rich meal

Food	Portion size (g)	Energy (kcal)	Protein (g)	Lipid (g)	Carbohydrate (g)	Fiber (g)
Butter	25	187	-	20.7	-	-
Sausage	50	139	8	11.6	2.5	-
Egg	50	73	6	5	1	-
Oat	20	70	-	2.5	12	4
Total	145	469	14	39.8	15.5	4

On the day reserved for each test, participants were advised to attend the Endocrinology Testing Room at 7:00 am after 10 hours of fasting, including water restriction. An intravenous cannula was inserted into a forearm vein, and blood sampling was collected at baseline (0 minutes). They were instructed to consume the meal within 15 minutes, without water or any beverage. Blood sampling was collected at 30, 60, 120, and 180 minutes after the complete meal intake. During the 48-hour interval between each test, the participants received standard frozen meals at home to standardize the macronutrient and caloric intake between the tests.

### Biochemical measurements and indexes

Blood samples were collected in plastic tubes containing EDTA, immediately placed on ice, and centrifuged at 4 °C in 1,500 × *g* for 15 minutes. Then, plasma samples were divided into small aliquots and stored at −70 °C for posterior glucose measurements, insulin, total GLP-1, C-peptide, glucagon, and non-esterified fatty acids (NEFA). Plasma glucose and NEFA were determined through an enzymatic colorimetric method (Labtest, MG, Brazil, and Wako Chemicals, CA, USA, respectively). Blood samples (4 mL) for total GLP-1 and glucagon determination were collected into separate ice-chilled tubes containing 80 μL of Aprotinin (Sigma A6279) and 40 μL of Diprotin A 10 mM (Sigma I9759). The total GLP-1 (GLP1T-36HK), glucagon (GL-32K), insulin (HI-14K), and C-peptide (HCP-20K) levels were analyzed using liquid-phase radioimmunoassay (RIA) according to the manufacturer's instructions (Millipore Corporation, Burlington, MA, USA).

Additional biochemical measurements were quantified in the Central Laboratory Division of the University Hospital, School of Medicine, University of São Paulo. The following analytes – triacylglycerol (TG), total cholesterol, LDL-cholesterol, and HDL-cholesterol – were determined through commercial kits (Labtest Diagnóstica S.A., Lagoa Santa, MG, Brazil). VLDL-cholesterol was estimated using the Friedewald equation (TG/5). The measurement of HbA1c was assessed through high-performance liquid chromatography (HPLC).

The homeostatic model assessment of insulin resistance (HOMA-IR) was estimated by multiplying fasting glucose (mmol/L) and fasting insulin (μIU/mL) and dividing by 22.5 (13). The Matsuda-insulin sensitivity index (ISI) was determined by the equation 10,000/square root of [fasting glucose x fasting insulin] x [mean glucose x mean insulin] (14), using the data from the carbohydrate-rich meal test. The quantitative insulin sensitivity check index (QUICKI) was calculated as 1/(log fasting insulin [μIU/mL] + log fasting glucose [mg/dL]) (15). The adipose tissue insulin resistance index (Adipo-IR) was calculated by multiplying fasting insulin (μIU/mL) and fasting NEFA concentrations (mmol/L) (16). The insulinogenic Index (IGI), an index of insulin secretion, was calculated using the equation Δ insulin (μIU/mL) 0-30 min/Δ glucose (mg/dL) 0-30 min, during the carbohydrate-rich meal test (17).

### Monitoring of participants

During the study, all participants were monitored and had at least 4 medical appointments with the endocrinologist and 2 appointments with the nutritionist. Additionally, a nurse with special training in diabetes maintained close contact with participants by phone. All of the participants were requested to control their blood glucose levels using a capillary glucometer, and they were instructed to call the researcher by phone whenever there was a change of > 200 mg/dL in either fasting or postprandial conditions, as well as in the case of other relevant signs or symptoms.

The participants were oriented to maintain their usual diet to avoid body weight alteration throughout the study period. Similarly, they were requested not to alter their usual physical exercise during the 4-month study period. Although no apparent residual medication effect was observed in the treated arm before they began the cross-over design (receiving the placebo), we did not register these data.

### Statistical analysis

Before statistical analysis, the symmetry of the data was verified with the Shapiro-Wilk test. Symmetric and asymmetric data were expressed as a mean ± standard deviation (SD) or median [interquartile ranges], respectively. The sitagliptin treatment effects were evaluated using a paired *t-*test (parametric) or Wilcoxon test (non-parametric). The effects of sitagliptin on plasma glucose, glucagon, insulin, C-peptide, total GLP-1, and NEFA levels during the MTTs were determined using a two-way repeated-measures ANOVA with Tukey's *post hoc* test. For comparison of delta values or area under the curve (AUC) data between different macronutrients, a one-way ANOVA was applied with Tukey's *post hoc* test. AUC and incremental AUC (iAUC) were calculated using the trapezoidal method directly through software tools. iAUC differed from the AUC, considering that the baseline values were discounted for all points evaluated. All described p values were two-sided, and p values < 0.05 were considered statistically significant. Data were analyzed using the GraphPad Prism (version 8.0.1).

## RESULTS

The analyses of the completers included 16 individuals, divided into two moments being n = 8 for each moment. Baseline participants’ characteristics are presented in Table 5. The mean age was 58 years (9 men and 7 women), and most of them were overweight or obese and with a family history of T2DM. The average values for plasma total-cholesterol, HDL-cholesterol, and LDL-cholesterol were mildly outside the reference values, while plasma insulin, glucagon, and NEFA were within the reference values. Plasma glucose, HbA1c, C-peptide, and triacylglycerol levels were higher than optimal values, characterizing the early state of a drug-naive individual with T2DM. No significant adverse effects were reported during the study.

**Table 5:** Characteristics of participants

Clinical characteristics	Baseline	After intervention	
		Placebo	Sitagliptin
Age (years)	58 ± 11		
Men/Women, n (%)	9/7 (56/44)		
BMI (kg/m^2^)	27 ± 2		
BMI > 25.0 kg/m^2^, n (%)	14 (87)		
Family history of T2DM, n (%)	11 (69)		
Systolic arterial hypertension, n (%)	9 (56)		
SAP (mmHg)	136 ± 26		
DAP (mmHg)	77 ± 16		
Total cholesterol (mg/dL)^a^	219 ± 52		
LDL-cholesterol (mg/dL)^a^	133 ± 50		
HDL-cholesterol (mg/dL)^a^,^b^	42 ± 13		
VLDL-cholesterol (mg/dL)^a^	33 [27–45]		
Triglycerides (mg/dL)^a^	165 [135–226]		
HbA_1c_ (%) (mmol/L)^a^	7.7 ± 1.2 (61 ± 0.1)		
Glucose (mg/dL)^a^	164 ± 43	137 [125–191]	130 [109–163]*
Insulin (μIU/mL)^a^	20 ± 14	19 ± 14	15 ± 8
C-peptide (ng/mL)^a^	3.8 ± 1.8	3.8 ± 1.8	3.6 ± 1.6
GLP-1 (pg/mL)^a^	719 ± 179	688 ± 209	689 ± 205
Glucagon (pg/mL)^a^	73 [57–88]	70 ± 17	74 ± 24
NEFA (mmol/L)^a^	0.61 ± 0.19	0.61 ± 0.19	0.58 ± 0.16
HOMA-IR		4.8 [2.2-10.1]	4.7 [2.6-7.6]
QUICKI		0.30 ± 0.03	0.31 ± 0.03
Adipo-IR		9.9 [5.7-14.9]	8.8 [6.0-10.6]
Matsuda index^c^		3 ± 2	3 ± 1.2
Insulinogenic index^c^		0.3 [0.1-0.65]	0.56 [0.28-0.73]*

Values are presented as mean ± SD or median [interquartile range] or number (n) followed by the respective proportion (%). n = 16.

* means p < 0.05 using paired Student *t*-test or Wilcoxon matched-pairs for symmetric and asymmetric data, respectively.

BMI: body mass index; T2DM: type 2 diabetes mellitus; SAP: systolic arterial pressure; DAP: diastolic arterial pressure; LDL: low-density lipoprotein; HDL, high-density lipoprotein; VLDL: very-low-density lipoprotein; HbA1c: haemoglobin A1c; GLP-1: glucagon-like peptide-1; NEFA: non-esterified fatty acids; HOMA: homeostasis model assessment of insulin resistance; QUICKI: quantitative insulin sensitivity check index; Adipo-IR: adipose tissue insulin resistance.

a Under fasting.

b No differences between man and women values.

c Insulinogenic index and Matsuda index were obtained from the CHO meal test.

### Gender-specific effects of sitagliptin treatment on glucose homeostasis parameters

The effects of sitagliptin treatment on glycemic and hormonal parameters are demonstrated in Table 5. Sitagliptin treatment reduced but did not normalize fasting plasma glucose levels (p < 0.05) and increased the insulinogenic index (p < 0.05). We observed no correlation between fasting plasma insulin and either plasma GLP-1 or NEFA levels after intervention with sitagliptin (Figures 1B and 1C). The delta (Δ) values for the Adipo-IR (obtained in relation to the respective gender-placebo values) were lower in men than in women, while the Δ IGI values were higher in men than in women, indicating augmented pancreatic beta-cell function, especially in men (Figures 1D, 1E, respectively).

### Plasma glucose, insulin, C-peptide, GLP-1, glucagon, and NEFA during CHO, PTN, and LIP meal tests

**Figure 2:**
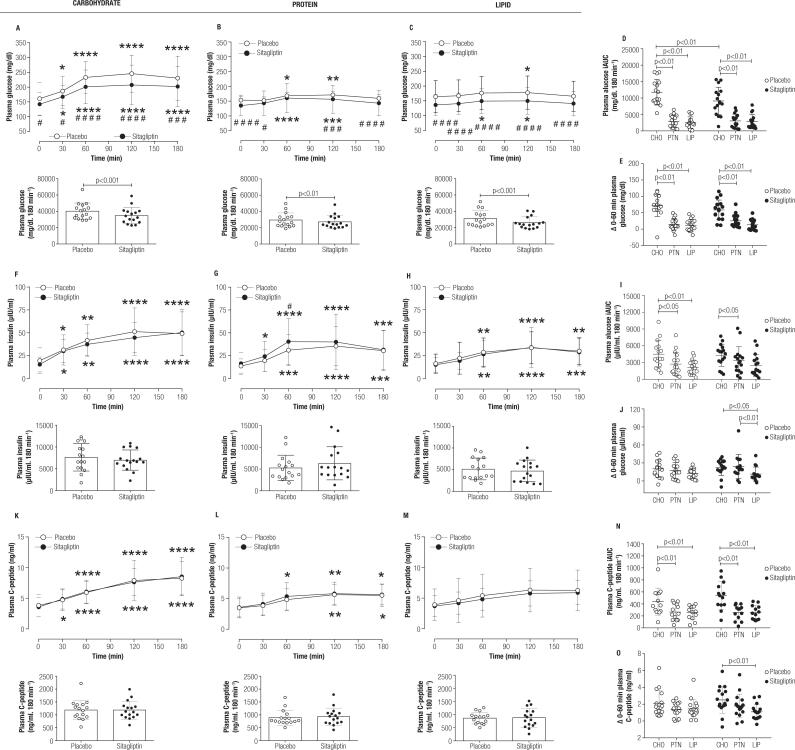
Effect of sitagliptin treatment on hormones and substrates during different MTTs. In **(A, F, K)** the plasma glucose, insulin and C-peptide values during a carbohydrate-rich meal tolerance test in placebo- and sitagliptin-treated (100 mg, *q.d.*, for 4 weeks) patients with early T2D and the respective AUCs. In **(B, G, L)** the plasma glucose, insulin and C-peptide values and their respective AUCs during a protein-rich meal tolerance test 48 h after the carbohydrate-rich meal tolerance test (same participants). In **(C, H, M)** the plasma glucose, insulin and C-peptide values and their respective AUCs during a lipid-rich meal tolerance test 48 h after the protein-rich meal tolerance test (same participants). In **(D, I, N)** the iAUC for plasma glucose, insulin and C-peptide, respectively, separated per macronutrient predominance. In **(E, J, O)** the Δ (0-60 min) values for plasma glucose, insulin and C-peptide, respectively, separated per macronutrient predominance. Data are mean ± SD. The statistical difference was obtained with 2-way RM for line graphs, paired Student *t*-test for AUC graphs **(A-C, F-H, K-M),** and 1-way ANOVA with Tukey *post hoc* test for scatter graphs **(D, E, I, J, N, O).** * Means significantly different from baseline values and ^#^ means significantly different from placebo group being * or ^#^ = p < 0.05, ** = p < 0.01, *** or ^###^ = p < 0.001 and **** or ^####^ = p < 0.0001. n = 16 for all graphs. AUC, area-under-the-curve; iAUC, incremental AUC, MTT, meal tolerance test; T2DM, type 2 diabetes mellitus; *q.d.*, from Latin *quaque die*, means once daily; CHO, carbohydrate-rich meal; PTN, protein-rich meal; LIP, lipid-rich meal.

Plasma glucose levels were lower during CHO, PTN, and LIP meal tests after sitagliptin treatment, except for min 60 in the PTN test. This observation was corroborated by lower plasma glucose AUC values after sitagliptin treatment for all conditions (Figures 2A-C). We registered the elevation of glycemia at minutes 30 and 60 during the CHO meal test, which remained higher than the baseline values up to the end of the study observation period (min 180), irrespective of placebo or sitagliptin treatments. As expected, the impact of PTN and LIP meals on glucose values was minimal, as observed with the correspondent iAUC and Δ 0-60 min data, irrespective of placebo or sitagliptin treatment (Figures 2D and 2E). Insulin and C-peptide achieved the highest values following the CHO meal test; however, insulin and C-peptide values did not differ between placebo and sitagliptin treatments (Figures 2 F-H and K-M), except for higher insulin values at min 60 in the PTN meal test after sitagliptin treatment. This observation was corroborated by the correspondent insulin and C-peptide iAUC data (Figures 2I and 2N). The Δ 0-60 min values for both insulin and C-peptide were higher in response to CHO *vs.* LIP meal tests after sitagliptin treatment (Figures 2J and 2O).

**Figure 3:**
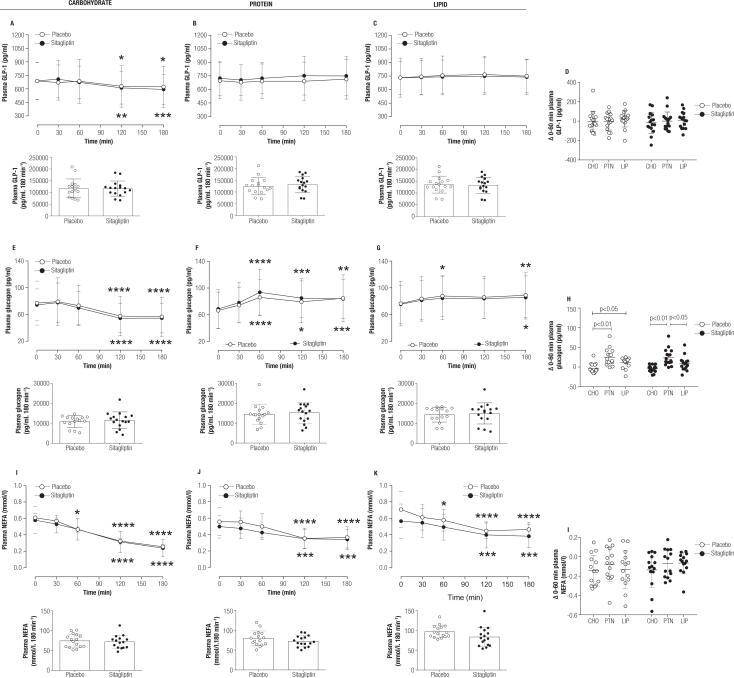
Effect of sitagliptin treatment on hormones and substrates during different MTTs. In **(A, E, I)** the plasma GLP-1, glucagon and NEFA values during a carbohydrate-rich meal tolerance test in placebo- and sitagliptin-treated (100 mg, *q.d.,* for 4 weeks) patients with early T2D and the respective AUCs. In **(B, F, J)** the plasma GLP-1, glucagon and NEFA values and their respective AUCs during a protein-rich meal tolerance test 48 h after the carbohydrate-rich meal tolerance test (same participants). In **(C, H, M)** the plasma GLP-1, glucagon and NEFA values and their respective AUCs during a lipid-rich meal tolerance test 48 h after the protein-rich meal tolerance test (same participants). In **(D, H, L)** the Δ (0-60 min) values for plasma GLP-1, glucagon and NEFA, respectively, separated per macronutrient predominance. Data are mean ± SD. The statistical difference was obtained with a 2-way RM for line graphs **(A-C, E-G, I-K),** and 1-way ANOVA with Tukey *post hoc* test for scatter graphs **(D, H, L).** * means significantly different from baseline values being * = p < 0.05, ** = p < 0.01, *** = p < 0.001 and **** = p < 0.0001. n = 16 for all graphs. AUC: area-under-the-curve; iAUC: incremental AUC; MTT: meal tolerance test; T2DM: type 2 diabetes mellitus; *q.d.*: from Latin *quaque die*, means once daily; GLP-1: glucagon-like peptide-1; NEFA: non-esterified fatty acids; CHO: carbohydrate-rich meal; PTN: protein-rich meal; LIP: lipid-rich meal.

Treatment with sitagliptin did not influence the total plasma GLP-1, glucagon, and NEFA values in any meal tested. No incremental GLP-1 values were observed during the first 60 min of meal tests (Figures 3A-D), but while total GLP-1 values were lower than baseline values 120 min after the CHO meal test, they remained sustained after PTN or LIP meal tests. Plasma glucagon values were lower than baseline values in the late period of the CHO meal test, while it became augmented from the min 60 in the PTN meal test and from min 180 in the LIP meal test in relation to baseline values, irrespective of placebo or sitagliptin treatment (Figures 3E-H). The levels of NEFA in the plasma became gradually reduced as the tests progressed, being more evident in response to the CHO meal test (Figures 3 I-L).

### Impact of sitagliptin treatment and macronutrient composition on functional aspects

The insulin and GLP-1 response following the CHO meal test effectively controlled overall plasma glucose after sitagliptin treatment as observed with higher insulin/glucose or GLP-1/glucose iAUC data, respectively (Table 6). However, treatment with sitagliptin did not influence the insulin/glucagon or insulin/NEFA AUC ratios. Considering the low glycemic and insulin responses to PTN and LIP meal tests *vs.* the CHO meal test, the hormonal ratios over plasma glucose were predominantly higher under PTN and LIP meal tests, while the insulin to either glucagon or NEFA ratios were predominantly lower during the PTN and LIP meal tests (Table 6).

**Table 6:** Impact of sitagliptin treatment and macronutrient composition on functional aspects

	Carbohydrate-rich meal			Protein-rich meal			Lipid-rich meal		
	Placebo	Sitagliptin	p	Placebo	Sitagliptin	p	Placebo	Sitagliptin	p
Insulin/glucose AUCs	0.2 ± 0.1	0.2 ± 0.1	ns	0.2 ± 0.1	0.3 ± 0.1	<0.01	0.2 ± 0.1	0.2 ± 0.1#	ns
Insulin/glucose iAUCs	0.4 ± 0.3	0.5 ± 0.3	<0.05	1.2 ± 0.9	1.8 ± 2.2*	ns	0.86 ± 0.47	1.3 ± 1.1	ns
GLP-1/glucose AUCs	10.1 ± 4.2	11.7 ± 4.1	<0.05	14.8 ± 5.6*	16.8 ± 5.5*	<0.05	15.3 ± 5.6*	17.4 ± 5.1*	<0.05
GLP-1/glucose iAUCs	1.5 ± 2.2	2.2 ± 3.5	<0.05	9.7 ± 16.2*	6.8 ± 10.4	ns	8.7 ± 12.0	6.5 ± 6.1	ns
C-peptide/glucose AUCs	0.03 ± 0.01	0.04 ± 0.01	<0.01	0.03 ± 0.01	0.04 ± 0.01	<0.05	0.03 ± 0.01	0.03 ± 0.01	ns
Insulin/glucagon AUCs	0.8 ± 0.5	0.7 ± 0.4	ns	0.4 ± 0.3*	0.5 ± 0.5*	ns	0.4 ± 0.2*	0.4 ± 0.4*	ns
Insulin/NEFA AUCs	106.3 ± 47.7	99.4 ± 32.8	ns	67.1 ± 30.4*	88.2 ± 52.2	<0.05	54.7 ± 28.5*	60.2 ± 33.4*	ns

Values are presented as mean ± SD. n = 16.

* p < 0.05 for the intergroup comparison (versus carbohydrate-rich meal).

# p < 0.05 for the intergroup comparison (versus protein-rich meal).

AUC: area under the curve; iAUC: incremental area under the curve; GLP-1: glucagon-like peptide-1; NEFA: non-esterified fatty acids.

## DISCUSSION

The agonists of GLP-1R are among the most effective drugs prescribed to treat T2DM, but their costs cannot be assessed, and they require injections. Despite being less effective than GLP-1R agonists, sitagliptin is an alternative oral drug to be considered when monotherapy (i.e., metformin) and lifestyle changes are not effective (18). In the present study, we showed that treatment of early T2DM patients with sitagliptin for 4 weeks achieved reduced fasting and post-meal plasma glucose values, independent of the predominant macronutrient composition in the ingested meal. Sitagliptin treatment also improved insulin/glucose and GLP-1/glucose iAUC ratios.

We expect the benefit of sitagliptin treatment to be associated with higher intact GLP-1 (active form) levels due to inactivation of DPP-4i, as already demonstrated in patients with T2DM after very short-term treatment with sitagliptin (19,20). However, the rise in intact GLP-1 during an MTT is not necessarily associated with the improvement of glucose, insulin and C-peptide values as observed in individuals with IFG treated with sitagliptin for 8 weeks (10). In our study, we did not observe a rise of total GLP-1 after the MTT, irrespective of macronutrient composition. The observation that total GLP-1 remained sustained during the entire period of PTN or LIP meal tests indicates that both luminal gut products of protein and lipid digestion exerted higher stimuli for GLP-1 secretion. However, we cannot be sure whether this is true for the intact GLP-1. The introduction of protein in a glucose tolerance test stimulates the release of other gut incretins, glucose-dependent insulinotropic peptide (GIP), in patients with overt diabetes for longer than 5 years (8). The demonstration that inhibition of GLP-1R with exendin-9 abolishes 50% of the sitagliptin action on lowering glucose values (7) suggests that GLP-1 must be involved in the reduced plasma glucose excursion in our study.

The effect of sitagliptin treatment upon insulin and C-peptide is more consistent when combined with metformin or mitiglinide (5,19). We did not find a major alteration in insulin or C-peptide by the effect of sitagliptin treatment, except for the higher insulin values at min 60 under the PTN meal test in relation to placebo. It has been known since the 1960s that protein load in a meal containing carbohydrates acts as a secretagogue for insulin (21), which was posteriorly confirmed for patients with T2DM (8). The higher iAUC values for insulin and C-peptide during the CHO meal test in relation to PTN and LIP meal tests agreed with the higher iAUC glucose values during the CHO meal test. This reiterates the concept that insulin secretion in early T2DM is not enough to normalize the plasma glucose values after a carbohydrate-rich meal and may indicate a possible reduction in insulin and C-peptide clearance in early T2DM. Hyperglycemia impairs insulin-induced insulin-degrading enzyme (IDE) activity in the liver cell model (22), and hepatic insulin clearance has been closely related to metabolic syndrome components (23). Thus, meals containing lower carbohydrates and higher protein or lipid content would benefit individuals with early T2DM by leading to lower daily blood glucose excursions and lower beta-cell demand, which can be welcomed for preserving beta-cell function.

Many diet interventions are indicated for the treatment of patients with T2DM, including low-carbohydrate, low-glycemic index, high-fiber, Mediterranean, and high-protein diets. A meta-analysis study demonstrated that the low-carbohydrate and Mediterranean diets led to more significant weight loss and an increase in HDL (24). Although no definitive consensus is established for whether a low-carbohydrate or low-fat diet works better for weight reduction, it was demonstrated that a low-carbohydrate diet works better for reducing vascular risk (25). The best diet would be one that works for each patient in sustaining reduction of body mass, and a reasonable way to be in adherence with the best diet practices is to prefer more unprocessed rather than ultra-processed food (26). Notwithstanding, the quality of diet is not the only factor that counts for the management of glucose homeostasis in T2DM but also the timing of food intake, as has been recently advocated (27).

Although sitagliptin treatment remained unchanged in glucagon excursion during the MTTs, the PTN meal test was the most effective in evoking higher plasma glucagon levels at min 60 in relation to baseline values, irrespective of whether sitagliptin or the placebo was administered. It is essential to mention that this peak of glucagon did not influence the glucose excursions that remained lower compared with the CHO meal test. After a protein-rich meal, the rise in glucagon is a counterregulatory response to avoid low glycemia after protein-induced insulin secretion (28).

Overall, sitagliptin treatment for 4 weeks was well-tolerated by drug-naive patients with T2DM and effectively reduced, but not normalized, fasting and post-meal glucose values. The insulinogenic index and the insulin/glucose, GLP-1/glucose iAUCs were also improved through sitagliptin treatment. Patients with early T2DM maintained lower glucose excursion after protein- or lipid-rich meals without any significant change in insulin, C-peptide, glucagon, or NEFA levels.

This study had some limitations. There was a lack of participants and non-participants to compare according to the main sociodemographic and clinical characteristics that could result in any type of bias, a lack of baseline clinical characteristics that could indicate any possible sitagliptin residual effect before the beginning of a cross-over design and a sample size since borderline significance in some comparisons could be attributed in part to the relatively small number of participants.

We conclude that the diet's carbohydrate, protein, and lipid content differently affects incretins (GLP-1), insulin, and glucagon. Finding therapeutic options that best fit the individuals’ dietary habits is part of the individualization of therapy in the treatment of T2DM. In drug-naive patients, sitagliptin effectively controls postprandial glycemia regardless of the composition of the diet, reinforcing its use as a therapeutic option for newly diagnosed patients with T2DM.
